# Targeting autophagy for postsynaptic organization and cognitive rescue in Fragile X syndrome

**DOI:** 10.4103/NRR.NRR-D-25-01685

**Published:** 2026-02-05

**Authors:** Cameron Keyser, Samantha J. Richardson, Jingqi Yan

**Affiliations:** Center for Gene Regulation in Health and Disease, Department of Biological, Geological, and Environmental Sciences, Cleveland State University, Cleveland, OH, USA

Fragile X syndrome (FXS) is the most common inherited form of intellectual disability and the leading monogenic cause of autism, accounting for 1%–6% of all autism cases in U.S. (Richter et al., 2015; Berry-Kravis et al., 2018; Hagerman and Hagerman, 2022). Patients with FXS exhibit complex and debilitating neurological phenotypes, including impaired cognition and social interactions, hyperactivity, attentional deficits, seizures, sleep disorders, and hypersensitivity (Berry-Kravis et al., 2018; Hagerman and Hagerman, 2022). FXS is caused by an expansion of a CGG trinucleotide repeat within the fragile X messenger ribonucleoprotein 1 (*Fmr1*) gene, leading to transcriptional silencing of *Fmr1* (Berry-Kravis et al., 2018; Hagerman and Hagerman, 2022). Fragile X Messenger Ribonucleoprotein (FMRP), the protein encoded by *Fmr1*, is an RNA-binding protein that controls the localization, stability, and translation of numerous RNAs essential for synaptic development, plasticity, and architecture (Richter et al., 2015). Decades of research have revealed that loss of FMRP causes synaptic deficits through multiple mechanisms, including exaggerated metabotropic glutamate receptors signaling, elevated translation of synaptic proteins, hypoactivity of inhibitory neurons, dysregulation of presynaptic plasticity, dysfunctional synaptic mitochondria, altered potassium channels, insufficient synaptic elimination, and downregulated autophagy (Berry-Kravis et al., 2018; Yan et al., 2018; Hagerman and Hagerman, 2022; Guo et al., 2023).

Based on these findings, therapeutic strategies have been developed to correct the molecular, synaptic, and behavioral deficits of FXS (Berry-Kravis et al., 2018; Hagerman and Hagerman, 2022). Among these, modulation of metabotropic glutamate receptor 5 pathway has shown promise in preclinical models. Enhancing GABAergic signaling represents another potential approach, with agents such as arbaclofen, a GABA_B_ agonist, exhibiting encouraging clinical outcomes. Pharmacological treatments with sertraline and cannabidiol are also being explored to alleviate behavioral and cognitive symptoms. A notable pharmacological treatment is metformin, a blood-brain barrier permeable type-II diabetes agent. Metformin activates AMP-activated protein kinase pathway, downregulating mammalian target of rapamycin and MEK-ERK signals, as well as supporting mitochondrial functions. Clinical trials have demonstrated promising results with metformin in both pediatric and adult FXS patients. Additionally, the PDE4D inhibitor BPN14770 has been shown to enhance cognition in adults with FXS, highlighting cAMP signaling as another therapeutic target. Recent gene reactivation approaches are aiming to reverse the silenced *Fmr1* expression and restore FMRP level. One such approach utilizes the CRISPR/Cas9 system. A targeted epigenetic editing using a dCas9–Tet1/CGG sgRNA system directly demethylates CGG repeats, restoring *Fmr1* expression and reducing FXS phenotypes in patient-derived neurons (Liu et al., 2018). Another emerging strategy aims to restore FMRP levels via antisense oligonucleotides (Shah et al., 2023). In FXS patients, *Fmr1* expresses an aberrant, mis-spliced mRNA isoform. By correcting mis-spliced *Fmr1* mRNA with antisense oligonucleotides, translation of FMRP is restored, highlighting a promising therapeutic approach for this disorder (Shah et al., 2023). Despite progress in these therapies, single target approaches often fail to address the broad symptomatology of FXS due to the disorder’s complex and multifactorial nature. Thus, future efforts are expected to focus on combinatorial or multi-pathway strategies.

The neuroanatomical hallmark of the FXS brain is the excess of immature synapses, which are causally related to cognitive, sensory, and social symptoms (Berry-Kravis et al., 2018; Hagerman and Hagerman, 2022). As the most abundant cytoskeletal protein in synapses, the filamentous form of actin (F-actin) is the primary structural support of synaptic stability and morphology. Proper regulation of actin polymerization (monomeric G-actin to F-actin) and depolymerization (F-actin to G-actin) is essential for correct synaptic formation, remodeling, and structural plasticity (Santini et al., 2017; Zhang et al., 2026). In FXS, dysregulated actin dynamics lead to F-actin overaccumulation, resulting in excessive immature synapses and contributing to cognitive and behavioral deficits (Santini et al., 2017; Zhang et al., 2026). These findings underscore the need to elucidate mechanisms underlying abnormal synaptic morphology and density.

Autophagy is a fundamental cellular process that degrades and recycles intracellular components (Palmer et al., 2025). In neurons, it plays key roles in protein turnover, axonal homeostasis, presynaptic vesicle release, and synaptic pruning (Yan et al., 2018; Palmer et al., 2025). Recent studies from our group and others have shown that neuronal autophagy is downregulated in FXS mouse models and patient-derived neurons (Yan et al., 2018; Guo et al., 2023). Thus, the goal of this study is to identify downstream protein targets of autophagy that mediate its regulatory effects on synaptic remodeling in normal conditions and, when dysregulated, lead to synaptic deficits in FXS (**Figure 1**). We hypothesize that, through degrading postsynaptic density protein 95 (PSD-95) and eukaryotic translation initiation factor 4 gamma 1 (eIF4G1) proteins, autophagy modulates Cofilin1 signal and postsynaptic cytoskeletal organization to affect synaptic stability and morphology, and that downregulated autophagy contributes to the synaptic and cognitive deficits observed in FXS (**Figure 2**).

**Figure 1:**
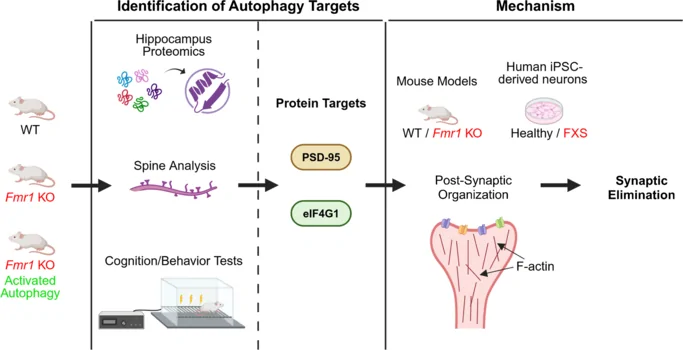
Schematic outline of the experimental design. Hippocampal tissues from WT mice, *Fmr1* KO mice, and *Fmr1* KO mice with activated autophagy were subjected to quantitative proteomic profiling. Follow-up analyses assessed dendritic spine density and morphology, along with behavioral tests of cognitive functions. These approaches identified eIF4G1 and PSD-95 as candidate protein targets mediating the effects of autophagy on synapses. Using both mouse models and human iPSC-derived neurons, mechanistic studies revealed that these proteins are crucial regulators of postsynaptic cytoskeletal organization and mediate autophagy-driven synaptic elimination. Created in BioRender. Keyser, C. (2025) https://BioRender.com/xzxm1bk. eIF4G1: Eukaryotic initiation factor 4G1; F-actin: filamentous actin; *Fmr1* KO: fragile X messenger ribonucleoprotein 1 knockout; FXS: fragile X syndrome; iPSC: induced pluripotent stem cells; PSD-95: postsynaptic density protein-95; WT: wild-type.

**Figure 2:**
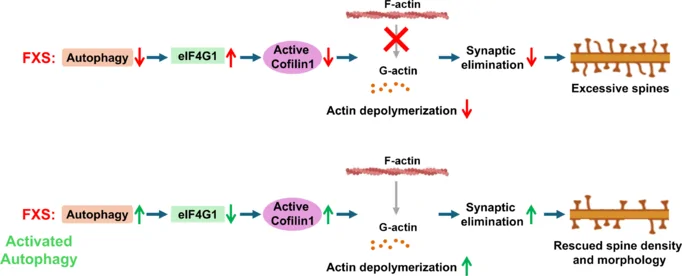
Autophagy regulates postsynaptic cytoskeletal organization and synaptic deficits in FXS. F-actin is the primary cytoskeletal protein in dendritic spines. In FXS, autophagy is downregulated, leading to increased eIF4G1, consequently lowering the level of active Cofilin1 protein, halting the depolymerization of F-actin to G-actin, which reduces synaptic elimination and increases synaptic density. When autophagy is activated, eIF4G1 is degraded, resulting in increased active Cofilin1, which promotes the depolymerization of F-actin, leading to proper synaptic elimination and rescue of synaptic density/morphology. Created in BioRender. Keyser, C. (2025) https://BioRender.com/v9k9o7b. eIF4G1: Eukaryotic initiation factor 4G1; F-actin: filamentous actin; FXS: Fragile X syndrome; G-actin: globular actin.

Individuals and animal models of FXS exhibit an overabundance of neuronal proteins that collectively disrupt signaling pathways and synaptic organization (Richter et al., 2015). Thus, we first profiled proteins that were upregulated in the hippocampus of *Fmr1* knockout (KO) mice, an animal model for FXS (**Figure 1**). Proteomic analysis identified 549 proteins significantly increased in the hippocampus of *Fmr1* KO mice *versus* wild-type controls. Of these 549 proteins, 289 were also upregulated in wild-type mice treated with chloroquine, an autophagy inhibitor, indicating that impaired autophagic degradation might crucially contribute to their accumulation. Gene Ontology analysis revealed that these 289 proteins are predominantly associated with postsynaptic organization, suggesting that defective autophagy plays a key role in FXS pathology and its downstream targets may mediate postsynaptic deficits.

To determine whether reduced autophagy critically contributes to the synaptic and cognitive deficits of FXS, we examined the effects of restoring autophagy in *Fmr1* KO mice (**Figure 1**). Administration of rilmenidine, an US Food and Drug Administration-approved, blood–brain barrier permeable autophagy activator, restored downregulated autophagy in hippocampal neurons and corrected the elevated spine density and immature synaptic morphology in *Fmr1* KO mice. Importantly, activating autophagy rescued cognitive deficits, improving performance in novel object recognition and contextual fear conditioning tasks. To test whether the rescue effects rely on activation of neuronal autophagy, *Fmr1* KO mice were crossed with *Atg7*^loxp^ mice and Syn1-Cre mice to generate *Fmr1* KO mice with neuron-specific deletion of ATG7, a key autophagy initiation protein. While rilmenidine effectively rescued synaptic and cognitive deficits in *Fmr1* KO mice, these effects were largely abolished in *Fmr1* KO mice lacking neuronal *Atg7*, indicating that rilmenidine’s therapeutic benefits critically depend on activating neuronal autophagy and highlighting a strong link between autophagic degradation and FXS pathology.

To elucidate how autophagy activation rescues synaptic and behavioral deficits, we performed further proteomic analysis to examine protein changes following rilmenidine treatment (**Figure 1**). Proteomic profiling of the hippocampus revealed that 42 proteins were elevated in *Fmr1* KO mice and in WT mice with inhibited autophagy, but reduced by rilmenidine treatment. These 42 proteins are enriched in synaptic and neuronal processes, including glutamate secretion and neuron morphogenesis. SFARI Gene database analysis identified that *Dlg4* (encoding PSD-95) and *Eif4g1* (encoding eIF4G1) among these 42 proteins are linked to autistic symptoms and reported with the highest number of autism cases. Notably, both PSD-95 and eIF4G1 were validated as direct autophagic degradation targets. Given the established role of PSD-95 as a postsynaptic scaffolding protein essential for postsynaptic stability and spine morphology (Yan et al., 2018), its autophagic degradation represents a key mechanism by which activation of autophagy rescues synaptic deficits in FXS. We next examined how eIF4G1 contributes to autophagy’s regulation on synapses. Results showed that autophagic degradation of eIF4G1 released eukaryotic translation initiation factor 4E (eIF4E) from eIF4G1/eIF4E interaction in *Fmr1* KO neurons, freeing eIF4E to sequester CYFIP1 (cytoplasmic FMR1 interacting protein 1), a critical component of Rac1-WAVE complex. This inhibited Rac1–WAVE assembly, decreased Cofilin1 phosphorylation, and promoted F-actin depolymerization, thereby reducing synaptic density and normalizing morphology (**Figure 2**). This mechanism reveals a direct molecular link between autophagic protein degradation and synaptic cytoskeletal organization, offering new insights into the pathophysiology of FXS.

The limited progress in FXS clinical trials largely stems from the gap between animal models and humans (Berry-Kravis et al., 2018; Hagerman and Hagerman, 2022). To assess the translational relevance of our research, we next validated our findings with neurons derived from human FXS induced pluripotent stem cells. Consistent with results from mouse models, human FXS neurons showed p62 accumulation, indicating reduced autophagy, along with elevated PSD-95 and eIF4G1 levels. Activation of autophagy effectively reduced p62 accumulation and decreased PSD-95 and eIF4G1 levels. Moreover, rilmenidine treatment normalized the Cofilin1 hyperphosphorylation and reduced F-actin to near-control levels. Thus, rilmenidine effectively restored autophagy and actin dynamics in human FXS neurons, supporting autophagy activation as a promising therapeutic strategy for FXS.

In summary, our study identifies a novel role of autophagy in regulating actin assembly, postsynaptic cytoskeletal organization, spine morphology, and cognition in FXS (Zhang et al., 2026). Activation of autophagy in hippocampal neurons promotes the degradation of PSD-95 and eIF4G1, thereby restoring actin dynamics, eliminating immature synapses, and improving cognition. Pharmacological activation of autophagy with rilmenidine significantly rescued cognitive deficits by normalizing actin assembly and spine structure. In addition to regulating actin dynamics, eIF4G1 also mediates cap-dependent translation (Zhang et al., 2026). Our results demonstrate that autophagy activation reduces overall protein synthesis by **~**20% in *Fmr1* KO neurons, indicating that eIF4G1 degradation may additionally influence synaptic plasticity through translational suppression. Because FMRP loss broadly impacts multiple pathways, impaired autophagy likely accounts for only part of the FXS phenotypes. Indeed, some behavioral deficits of *Fmr1* KO mice, such as nest building and open-field performance, were not rescued by autophagy activation. Although rilmenidine has shown no significant effects on body weight or growth in our study, caution is still needed when considering rilmenidine as a therapeutic option for FXS in the future, because systemic administration of rilmenidine has been reported to potentially affect many other tissues in addition to the brain. Collectively, our findings establish autophagy as a potent, multitarget therapeutic strategy for synaptic and cognitive deficits in FXS. Targeting autophagy thus represents a promising approach for correcting core synaptic dysfunctions in FXS and potentially other autism spectrum disorders.

*This work was supported by The National Institutes of Health grant NS118378, the Cleveland State University Startup grant, and the NARSAD Young Investigator Grant (#28792) to JY.*

## References

[R1] Berry-Kravis EM, Lindemann L, Jonch AE, Apostol G, Bear MF, Carpenter RL, Crawley JN, Curie A, Des Portes V, Hossain F, Gasparini F, Gomez-Mancilla B, Hessl D, Loth E, Scharf SH, Wang PP, Von Raison F, Hagerman R, Spooren W, Jacquemont S (2018). Drug development for neurodevelopmental disorders: lessons learned from fragile X syndrome. Nat Rev Drug Discov.

[R2] Guo Y, Shen M, Dong Q, Mendez-Albelo NM, Huang SX, Sirois CL, Le J, Li M, Jarzembowski ED, Schoeller KA, Stockton ME, Horner VL, Sousa AMM, Gao Y, Birth Defects Research L, Levine JE, Wang D, Chang Q, Zhao X (2023). Elevated levels of FMRP-target MAP1B impair human and mouse neuronal development and mouse social behaviors via autophagy pathway. Nat Commun.

[R3] Hagerman RJ, Hagerman PJ (2022). Fragile X syndrome: lessons learned and what new treatment avenues are on the horizon. Annu Rev Pharmacol Toxicol.

[R4] Liu XS, Wu H, Krzisch M, Wu X, Graef J, Muffat J, Hnisz D, Li CH, Yuan B, Xu C, Li Y, Vershkov D, Cacace A, Young RA, Jaenisch R (2018). Rescue of fragile X syndrome neurons by DNA methylation editing of the FMR1 gene. Cell.

[R5] Palmer JE, Wilson N, Son SM, Obrocki P, Wrobel L, Rob M, Takla M, Korolchuk VI, Rubinsztein DC (2025). Autophagy, aging, and age-related neurodegeneration. Neuron.

[R6] Richter JD, Bassell GJ, Klann E (2015). Dysregulation and restoration of translational homeostasis in fragile X syndrome. Nat Rev Neurosci.

[R7] Santini E, Huynh TN, Longo F, Koo SY, Mojica E, D’Andrea L, Bagni C, Klann E (2017). Reducing eIF4E-eIF4G interactions restores the balance between protein synthesis and actin dynamics in fragile X syndrome model mice. Sci Signal.

[R8] Shah S, Sharp KJ, Raju Ponny S, Lee J, Watts JK, Berry-Kravis E, Richter JD (2023). Antisense oligonucleotide rescue of CGG expansion-dependent FMR1 mis-splicing in fragile X syndrome restores FMRP. Proc Natl Acad Sci U S A.

[R9] Yan J, Porch MW, Court-Vazquez B, Bennett MVL, Zukin RS (2018). Activation of autophagy rescues synaptic and cognitive deficits in fragile X mice. Proc Natl Acad Sci U S A.

[R10] Zhang Z, Keyser C, Li Y, Rosolia BJ, Porch MW, Zhang W, Su B, Jiang P, Zukin RS, Yan J (2026). Autophagy controls the hippocampal postsynaptic organization and affects cognition in a mouse model of Fragile X syndrome. Mol Psychiatry.

